# The Role of Compassionate Technology in Blended and Digital Mental Health Interventions: Systematic Scoping Review

**DOI:** 10.2196/42403

**Published:** 2023-04-07

**Authors:** Charlotte van Lotringen, Benedetta Lusi, Gerben J Westerhof, Geke D S Ludden, Hanneke Kip, Saskia M Kelders, Matthijs L Noordzij

**Affiliations:** 1 Department of Psychology, Health and Technology University of Twente Enschede Netherlands; 2 Department of Design, Production and Management University of Twente Enschede Netherlands

**Keywords:** compassionate technology, digital mental health interventions, eHealth, e–mental health, compassion, mental health care

## Abstract

**Background:**

An essential value in mental health care is compassion: awareness of suffering, tolerating difficult feelings in the face of suffering, and acting or being motivated to alleviate suffering. Currently, technologies for mental health care are on the rise and could offer several advantages, such as more options for self-management by clients and more accessible and economically viable care. However, digital mental health interventions (DMHIs) have not been widely implemented in daily practice. Developing and evaluating DMHIs around important mental health care values, such as compassion, could be key for a better integration of technology in the mental health care context.

**Objective:**

This systematic scoping review explored the literature for previous instances where technology for mental health care has been linked to compassion or empathy to investigate how DMHIs can support compassion in mental health care.

**Methods:**

Searches were conducted in the PsycINFO, PubMed, Scopus, and Web of Science databases, and screening by 2 reviewers resulted in 33 included articles. From these articles, we extracted the following data: technology types, goals, target groups, and roles of the technologies in the intervention; study designs; outcome measures; and the extent to which the technologies met a 5-step proposed definition of compassion.

**Results:**

We found 3 main ways in which technology can contribute to compassion in mental health care: by showing compassion to people, by enhancing self-compassion in people, or by facilitating compassion between people. However, none of the included technologies met all 5 elements of compassion nor were they evaluated in terms of compassion.

**Conclusions:**

We discuss the potential of compassionate technology, its challenges, and the need to evaluate technology for mental health care on compassion. Our findings could contribute to the development of compassionate technology, in which elements of compassion are explicitly embedded in its design, use, and evaluation.

## Introduction

### Background

Currently, digital technologies for mental health care are on the rise [1,2]. Examples include not only internet-delivered cognitive behavioral therapy (CBT) but also virtual reality (VR) and artificial intelligence–enabled programs [3], mobile apps [4], socially assistive robotics [5], and serious games [6,7]. Given the increasing costs of care and growing waiting lists in this field, technology could be an important element of sustainable mental health care [3]. Technology offers a wealth of possibilities to complement health care professionals by taking over certain tasks, lightening their workload, and providing innovative ways to deliver health care [8]. In addition, technology can make mental health care more accessible to clients, give them more insight into their own mental health, and offer flexibility through personalization [9,10]. Digital mental health interventions (DMHIs) can be used as stand-alone interventions or in blended treatments: combining *traditional* face-to-face psychotherapy techniques with digital interventions [11]. Blended treatment has the potential to offer the *best of both worlds*, as it could be used to better match the intensity of the treatment to the severity of a client’s complaints and to enhance a client’s self-management [12].

Many forms of DMHIs exist and are shown to be effective in clinical trials [13], and if used well, they can often be as effective as face-to-face treatments [14-17]. However, the acceptance and use of these technologies remain low among both clients and professionals [18,19]; therefore, the actual implementation of DMHIs in daily practice has been limited. Among clients, there are low levels of adherence to DMHIs [20,21]. A potential reason for this could be that current DMHIs are often very direct translations of nondigital interventions, such as web-based CBT interventions, which closely follow their evidence-based nondigital versions [22]. Owing to the larger focus on content (eg, established therapeutic techniques) than on the user (eg, how the user prefers to engage with them), DMHIs are often perceived as impersonal [22]. This seems to be a missed opportunity, given that allowing for personalization is one of the advantages that technology could offer. Thus, the possibilities technology offers are not being optimally used. A further challenge is that professionals show hesitation and a lack of digital skills to implement DMHIs [18]. Similar to what we see from the client’s perspective, this hesitation in part stems from the doubt whether personal, empathic connections can be made or supported if DMHIs are used [18]. Moreover, professionals indicate that an explicit conceptual foundation for the use of DMHIs is currently lacking, meaning that it is not clear, and at best implicit, why and how DMHIs can be embedded in the mental health care system [19].

### Compassionate Technology

Given these current barriers, a new conceptual foundation is needed to build a bridge between technology and the context of mental health care. This could be done by expanding our view of the design, use, and evaluation of DMHIs to include and center on the values that are foundational to mental health care. Values are personal or societal judgments of what is valuable and important in life [23]. Compassion is widely recognized as central and essential in mental health care or health care in general [24-26]. In short, compassion refers to the awareness of suffering and motivation to act to alleviate suffering. Although conceptions and practices surrounding compassion have existed in Buddhism for >2500 years, in Western psychology, the construct of compassion has become a topic of study only more recently [27].

After comparing and synthesizing earlier definitions and measures of compassion from science, religion, and health care, Strauss et al [28] proposed that compassion is a cognitive, affective, and behavioral process containing five elements: (1) recognizing suffering, (2) understanding the universality of suffering in the human experience, (3) feeling empathy for the person suffering and connecting with the distress (emotional resonance), (4) tolerating one’s own uncomfortable feelings that arise in the face of suffering (eg, distress, anger, and fear), to remain open and accepting of the person suffering, and (5) acting or feeling motivated to alleviate suffering [28].

Compassion can be directed not only toward ourselves (often referred to as self-compassion [29]) and loved ones but also toward strangers and ultimately toward all humankind [30].

Although empathy is part of the compassionate elements, compassion is conceptually different from empathy. Empathy has been defined as the vicarious experience of another’s emotions [31], where one understands, is affected by, and shares another’s emotions and perspectives [32]. It does not involve a motivation to act to alleviate another person’s suffering. More specifically, although empathy can also lead to behavioral outcomes, it is not part of the concept of empathy itself [33]. In contrast, the process of compassion is specifically a response to suffering and not to emotions in general. It not only entails connecting with another’s distress but also understanding the universality of suffering in the human experience and tolerating uncomfortable feelings that can be aroused in response to the suffering person, so that one can remain open and accept this person and then act to alleviate their suffering [28]. The ability to tolerate uncomfortable feelings is essential for preventing empathic distress. Empathic distress occurs when one is more upset *by* another’s suffering than one is concerned *for* the other [34]. It can lead people to close themselves off from suffering and tends to inhibit compassion [35,36]. Thus, compassion contains elements that are specifically relevant to mental health care, more so than empathy alone.

Embedding compassion in health care improves clinical outcomes, perceived quality of care, and patient satisfaction; strengthens the therapeutic alliance; and supports patient-centered care [28,37,38]. Although research is slowly beginning to include compassion as a factor in technology in general [39], research on the link between compassion and technology for mental health care is still scarce. An exception is a recent scoping review by Kemp et al [37], who investigated how digital technologies were being used by patients and professionals in the delivery of compassionate mental health care. In addition, it investigated the facilitators and barriers for the use of digital technology in the delivery of compassionate mental health care. The authors found that when used appropriately, digital technologies can facilitate and strengthen compassion and meaningful human connections in mental health care. Moreover, technology can create new means for relationships between mental health professionals and patients. Kemp et al [37] focused their review on compassionate care and examined DMHIs with a model of digital intersections with compassionate care [40]. In this review, we take a different but complementary approach, examining DMHIs with the elements of compassion in itself [28]. This way, we aimed to explore how DMHIs could support the different components of compassion as a process. We expect this process-view of compassion could be a helpful and practical guiding force to shape compassionate blended treatment.

As compassion is a pivotal value in the mental health care context, emphasizing the process of compassion as a central value in the design, evaluation, and use of mental health care technology could be key to make it more suitable to the needs of clients and professionals. In turn, this could increase the uptake and integration of technologies in current treatments, ultimately ensuring compassionate blended mental health care that realizes its potential. On the basis of the proposed definition of compassion by Strauss et al [28], technology that is designed, developed, and evaluated around the value of compassion would enable and facilitate elements of compassion, or in short, the recognition and alleviation of suffering. To the best of our knowledge, this concept of *compassionate technology* for mental health care has not been systematically studied. This highlights the need for a clear conceptualization of compassionate technology based on research in which DMHIs have been explicitly linked to compassion.

### Research Objectives

This systematic scoping review provides an overview of how and to what degree elements of compassion have been linked to digital technologies for mental health or mental health care in previous studies. We used this information to describe the current status and scope of research on technology that is connected to compassion and to inform the future development of compassionate technology.

To reach these aims, we formulated the following research questions:

1. What types of technology for mental health care have been connected to compassion in previous studies, for what goals and which target groups were they developed, and what was the role of the technology in the intervention (eg, stand-alone or blended treatment)?
2. What study designs have previously been used to study these technologies, and what outcome measures are used?
3. To what extent and how do these technologies meet the 5 elements of compassion as distinguished by Strauss et al [28]?

## Methods

### Research Design

A systematic scoping review was conducted in accordance with existing guidelines [41]. This approach is particularly useful for bringing together the literature in disciplines with emerging evidence when a body of the literature has not yet been reviewed or exhibits a large, complex, or heterogeneous nature that is not suitable for a more precise systematic review [41]. Because of the novelty of the field and the presumed limited previous research on technologies that foster compassion, a systematic scoping review was deemed the appropriate method, as it focuses on appraising a body of literature on a specified topic in terms of extent, range, and nature [42].

### Search Strategy

The search strategy was developed iteratively in consultation with an information specialist, as suggested by Horsley [43]. A systematic search was conducted between October and November 2020 using the PsycINFO, PubMed, Scopus, and Web of Science databases. The 4 databases were chosen because PsycINFO focuses on research on behavioral science and mental health, PubMed focuses on biomedicine (including psychiatry), and the other 2 databases include research from all disciplines. In this way, psychology, psychiatry, and technology research fell within the scope of our search. The query used a combination of terms related to *compassion*, *technology*, and *mental health* occurring in the title, abstract, or keywords of published articles (see Multimedia Appendix 1 for the search strings). The search terms used were chosen to focus specifically on the mental health field as well as on explicit mentions of either compassion or the related term empathy.

### Eligibility Criteria

Because of the novelty of the subject, this review aims to identify articles covering any form of digital technology for the support of mental health that fosters the presence of compassion. Digital technologies linked to empathy instead of compassion were also eligible to avoid excluding articles that used the term “empathy” instead of compassion while pertaining to a similar construct. Our review did not focus on one specific type of participant in the included studies, so that articles including different types of people in and around the mental health field were eligible (eg, therapists, clients or patients, [informal] caregivers and also the public). All types of original research studies were eligible from any year of publication, country of origin, or original language (if a copy was available in English, German, Italian, or Dutch).

Exclusion criteria were as follows:

1. Articles without a clear focus on compassion or empathy, for example, merely mentioning compassion or empathy without further elaboration or using these factors solely as predictors or outcomes in a study.
2. Articles in which the link with compassion or empathy was *only* found as part of web-based versions of established compassion-based approaches (eg, Compassion-Focused Therapy [44]) or in the delivery of written psychoeducation (eg, a website with information on self-compassion), and compassion or empathy did not refer to (interactions with or through) the technology itself. These articles were excluded because a lot of research has already been conducted on these therapeutic approaches [45], and the role technology plays is relatively small, so that it is not directly relevant for our current purposes.
3. Articles related to compassion or empathy in a different field than mental health, for example, education or health care in general without a focus on mental health.

### Study Selection

**Figure 1:**
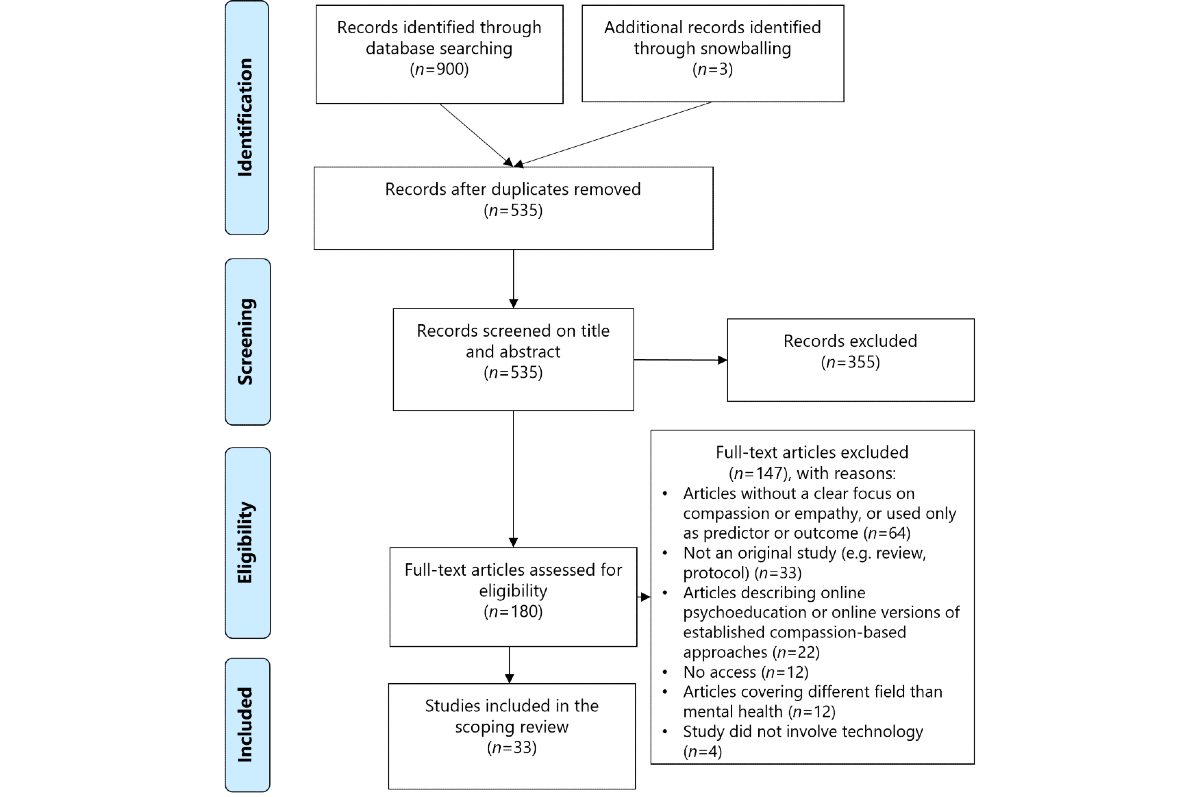
PRISMA (Preferred Reporting Items for Systematic Reviews and Meta-Analyses) flowchart of the selection process for the systematic scoping review.

Covidence (a literature review screening software recommended by Cochrane [46]) was used to filter duplicate articles and facilitate study selection in 3 steps (Figure 1). First, the titles and abstracts of all retrieved articles were screened for eligibility by 2 authors (CvL and BL). Disagreements on the inclusion or exclusion of papers were discussed until an agreement was reached. Second, the full text of all remaining articles was checked for inclusion by one author (CvL) and doubts were discussed with a second author (BL). If an agreement could not be reached between the 2 authors, a third researcher was consulted (MLN). Third, to check whether seminal works were overlooked during the initial search process, forward and backward snowballing by one author (CvL) was used, based on the reference lists and citations of the included papers.

### Data Extraction

#### Overview

Data that were extracted from each included article were author or authors, country of origin, year of publication, type of technology, target group or groups, goal or goals of the technology, the role of technology in the intervention, study design, outcome measures, elements of compassion, and compassionate role of technology. The role of technology in the intervention was coded depending on whether a technology was used by the target group on their own without guidance (“stand-alone”); *only* to deliver treatment sessions with a therapist or coach (“telecommunication”); with guidance from a coach or therapist, which was not face-to-face (“guided”); or was integrated into a treatment with face-to-face therapy sessions (“blended treatment”). Study designs were classified as qualitative, quantitative cross-sectional, quasi-experimental, experimental, or a combination (adapted from Centre for Evidence-Based Medicine [47]), with quasi-experimental studies referring to nonrandom allocation to groups and experimental studies referring to random allocation. For consistency, the term “outcome measures” was used for both quantitative and qualitative study designs, in the latter cases referring to the investigated variables. Elements of compassion were first described by closely following the author’s wording and then coded in the matching compassionate element (recognition, universality, empathy, tolerance, acting) by comparing them to their descriptions in Strauss et al [28]. Thus, the final results table includes the coded compassionate element and how this was described in the included study.

As we noticed that mentions of empathy or compassion in the included articles referred to different processes that technology could support, we divided articles into 3 “compassionate roles,” used to structure our results. These compassionate roles of technology have been categorized into different codes through inductive coding [48] using the method of constant comparison [49]. The roles were coded depending on whether the mentions of compassionate elements were mainly used to describe the features of the technology itself (Role A), to describe interventions for self-compassion (Role B), or to describe technology to facilitate compassionate elements between people (Role C). The characteristics of all included studies were extracted by 1 author (CvL), and data extraction of 15% of the included studies was validated by a second author (BL) with 82% agreement. This percentage of agreement was deemed sufficient to continue data extraction by one author. Data extraction occurred iteratively in consultation with several of the coauthors, where doubts were discussed until consensus was reached.

#### Main Perspective for Qualitative Analysis

In qualitative research, it is valuable to consider and describe the perspective held by researchers and how this could have influenced the research [50,51]. Therefore, we provide a short description of the background and perspective of the first author (CvL), focusing on elements that might have influenced the research process and vision presented in this paper.

CvL is a Dutch woman born in the Netherlands and raised in a nonreligious environment. Compassionate technology for mental health care is the topic of her PhD research project. The aim of this project was to investigate how technology in mental health care can be integrated into daily mental health care practice, where compassion is a fundamental value. This project took place in collaboration with researchers from different disciplines, a mental health care organization, and an eHealth company. CvL’s prior understanding of the topic comprises literature research on compassion, technology, and mental health care, as well as interviews with mental health care professionals, clients, and developers of DMHIs and observations of ways of working of mental health care professionals. Compassion is also an important personal value to her, in the sense of feeling responsibility for the world around you and wanting to contribute positively to society.

## Results

### Overview

**Figure 2:**
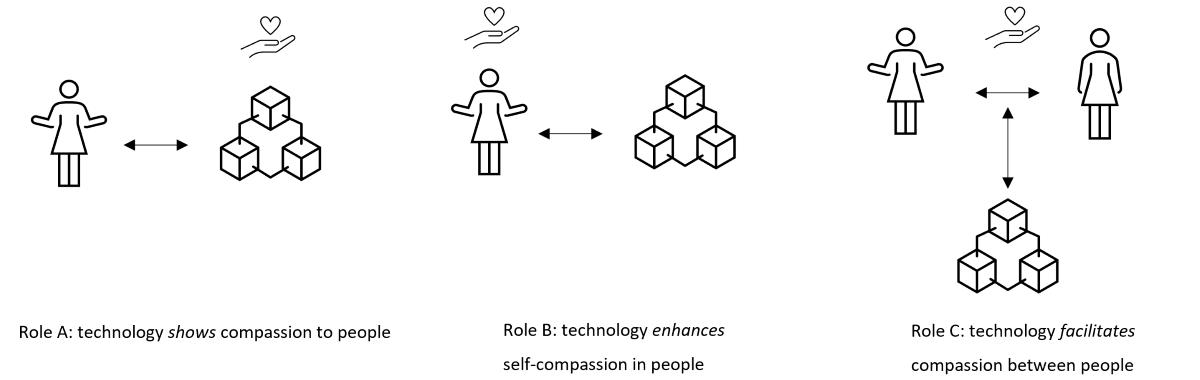
Schematic display of the 3 identified roles in which technology can contribute to compassion.

On the basis of the inclusion and exclusion criteria, we included 33 studies (Tables 1-3), covering 31 unique DMHIs. These studies were published between 2008 and 2020, with the majority published between 2016 and 2020 (25/33, 76%) and conducted in Western countries (31/33, 94%). The technologies we found could be divided into 3 roles based on the main way the technology contributed to the presence of compassion (Figure 2). The technology could show elements of compassion *to* people (Role A, n=8), technology could enhance elements of self-compassion *in* people (Role B, n=12), and technology could facilitate elements of compassion *between* people (Role C, n=13).

**Table 1:** Extracted data from the included studies classified as Role A: technology shows elements of compassion to people (n=8).

Role and study			Technology (technology, target group, goal, and role in intervention)		Study (study design and outcome measures)		Compassionate elements (on the basis of the 5 elements proposed by Strauss et al [28])
	Hauser-Ulrich et al [52], 2020, Switzerland	Technology: mobile health intervention using a fully automated text-based health care chatbot (SELMA) Target group: patients with ongoing or cyclic pain Goal: to deliver personalized intervention modules for mental health and psychoeducation for pain management, and to build a working alliance between a participant and the chatbot Role in intervention: stand-alone		Design: experimental (pilot RCT^a^) Outcome measures: symptom and well-being outcomes, working alliance, adherence, and acceptability		Recognition^b^: chatbot enquires about the participants’ mood Empathy^c^: chatbot replies in an empathic way, and is also experienced as such by participants Acting^d^: chatbot addresses participants’ accountability by supporting the completion of activities and tasks and motivating participants to repeat them	
	Morris et al [53], 2018, United States	Technology: text-based conversational agent in a peer-to-peer platform, that draws and repurposes responses from a pool of peer-support data (Koko) Target group: users of Koko Goal: to express empathic support Role in intervention: stand-alone		Design: qualitative and experimental Outcome measures: ratings of the conversational agent’s responses (“good, okay, bad”)		Empathy: conversational agent gives a nuanced and empathic reply addressing specific elements of the user’s message	
	Javed and Park [54], 2019, United States	Technology: socially assistive robot Target group: children with autism spectrum disorder Goal: to regulate the user’s emotions and prime higher social engagement in a user Role in intervention: stand-alone		Design: qualitative Outcome measures: engagement in intervention		Recognition: the user selects their current emotion state from an input panel Empathy: after the user indicated their current emotion state, the robot’s initial display of emotions closely follow the user’s Acting: the robot’s subsequent emotion selections lead the user toward a desired (goal) emotional state, to support emotion regulation	
	Pontier and Siddiqui [55], 2008, Netherlands	Technology: web-based agent Target group: people filling in the BDI^e^ Goal: to guide the user through the BDI and respond empathically to the answers given by the user Role in intervention: stand-alone		Design: quasi-experimental Outcome measures: ratings of how friendly, interested, trustworthy, and kind the web-based agent was		Empathy: the web-based agent responds to the answers given on the BDI by the user, either by showing happiness or a neutral expression when the user seems fine, or sadness when the user appears to be more depressed	
	Kohori et al [56], 2018, Japan	Technology: interactive therapy robot Target group: people who have difficulty living with pets Goal: to induce a “healing mood” Role in intervention: stand-alone		Design: quantitative cross-sectional and qualitative Outcome measures: design elements for inducing a healing mood, acceptability		Empathy: the robot can recognize and track human faces and nod while someone is talking	
	Fitzpatrick et al [57], 2017, United States	Technology: fully automated conversational agent, desktop or mobile accessible (Woebot) Target group: college students self-reporting symptoms of anxiety and depression Goal: to deliver a self-help program in a convenient and engaging way Role in intervention: stand-alone		Design: experimental (RCT) Outcome measures: symptom and well-being outcomes, acceptability, and usability		Recognition: participants could indicate their mood, and received a weekly mood description Empathy: the bot replied in an empathic way, appropriate to participants’ inputted mood Acting: the bot presented CBT^f^-based content based on users’ mood state, asked for and checked up on users’ personal goals and sent personalized messages for motivation	
	Bickmore et al [58], 2010, United States	Technology: animated conversational agent Target group: patients with schizophrenia Goal: to promote antipsychotic medication adherence, physical activity, system use and foster the therapeutic alliance Role ins intervention: stand-alone		Design: quasi-experimental (pilot evaluation study) Outcome measures: system use and interaction times, medication adherence, and physical activity		Empathy: the agent responds empathically, by expressing verbal and nonverbal concern for a negative state of the user Acting: agent asks about medication-taking behavior, provides feedback based on self-monitoring charts, and reminds user of goal behavior and when to get medication refills	
	Rouaix et al [59], 2017, France	Technology: humanoid robot (NAO), with Wizard of Oz approach Target group: people with dementia Goal: to act as therapist’s assistant in psychomotor therapy, as a mediating tool Role in intervention: blended treatment		Design: experimental Outcome measures: well-being outcomes, engagement, satisfaction with intervention, appreciation of robot, and empathy-related behaviors in human-robot interaction		Recognition: robot is able to acknowledge the participants emotions and feelings Empathy: robot exhibits empathic gestures such as giving confirmation signs by head movements Acting: robot gives positive feedback and acknowledges participant’s performance to boost confidence and motivation	

^a^RCT: randomized controlled trial.

^b^Recognizing suffering.

^c^Feeling empathy for the person suffering and connecting with the distress (emotional resonance).

^d^Motivation to act or acting to alleviate suffering.

^e^BDI: Beck Depression Inventory.

^f^CBT: cognitive behavioral therapy.

**Table 2:** Extracted data from the included studies classified as Role B: technology enhances elements of self-compassion in people (n=12).

Role and study			Technology (technology, target group, goal, and role in intervention)		Study (study design and outcome measures)		Compassionate elements (on the basis of the 5 elements proposed by Strauss et al [28])
	Ascone et al [60], 2020, Germany	Technology: immersive compassion-focused imagery VR^a^ intervention Target group: student sample with slightly elevated symptoms of paranoia Goal: to enable a sense of presence and induce specific emotional responses to support the development of self-compassionate feelings Role in intervention: stand-alone		Design: experimental Outcome measures: symptom and well-being outcomes, self-compassion, and self-rated intervention benefit		Universality^b^: the VR intervention was set in space, to evoke an overview effect and elicit feelings of connectedness with humanity and self-transcendence Acting^c^: participants were guided into opening for feelings of kindness, warmth, wisdom, courage, and strength while also interacting with a space nebula representing compassion, which reacted to touch by intensifying its glow	
	Brown et al [61], 2020, United Kingdom	Technology: VR with compassionate coach imagery Target group: individuals scoring highly for paranoia Goal: to reduce negative beliefs about the self, and hence paranoia Role in intervention: stand-alone		Design: experimental (RCT^d^) Outcome measures: symptom and well-being outcomes and self-compassion		Empathy^e^: participants created a personal compassionate coach who provided strength, kindness, and warmth Acting: the compassionate coach helped participants to feel better able to cope with everyday challenges and to be more self-compassionate	
	Pennesi and Wade [62], 2018, Australia	Technology: web-based imagery rescripting intervention Target group: body-dissatisfied young women at risk of developing an eating disorder Goal: to reduce disordered eating by strengthening protective factors (ie, self-compassion) Role in intervention: stand-alone		Design: experimental (RCT) Outcome measures: symptom and well-being outcomes and self-compassion		Empathy: patients were asked to imagine and write about their earliest memory of an unpleasant body experience from both an observers’ perspective and in the present with their wiser and more compassionate self in the room	
	Falconer et al [63], 2016, United Kingdom	Technology: VR Target group: patients with major depressive disorder Goal: to facilitate self-compassion through embodiment, also for people who find it difficult to be self-compassionate Role in intervention: stand-alone		Design: quasi-experimental Outcome measures: symptom and well-being outcomes, self-compassion, and “VR experience” (eg, feeling of being comforted)		Acting: participants were provided with sentences to reduce distress based on compassion-focused therapy and asked to deliver them compassionately to a virtual child and then experienced them from the perspective of the child	
	Falconer et al [64], 2014, United Kingdom	Technology: VR Target group: healthy females high in self-criticism Goal: to foster self-compassion through embodiment Role in intervention: stand-alone		Design: experimental Outcome measures: symptom and well-being outcomes and “VR experience”		Acting: participants were provided with sentences to reduce distress based on compassion-focused therapy and asked to deliver them compassionately to a virtual crying child, and then experienced them from the perspective of the child	
	Donovan et al [65], 2016, United States	Technology: mobile phone app (BodiMojo) Target group: adolescents Goal: to foster coping and well-being during adolescence Role in intervention: stand-alone		Design: quantitative and qualitative Outcome measures: use data and satisfaction		Recognition^f^: the app allows the user to track their feelings in a mood cloud, giving a visual representation of their mood and get personalized feedback Universality: daily wellness tips can be linked to emphasizing common humanity Acting: daily wellness tips prompt users to engage in mindfulness and self-compassion activities	
	Rodgers et al [66], 2018, United States	Technology: mobile phone app (BodiMojo) Target group: adolescents Goal: to promote positive body image and self-compassion Role in intervention: stand-alone		Design: experimental (RCT) Outcome measures: symptom and well-being outcomes and self-compassion		Recognition: the app allows the user to track their feelings daily in a mood cloud, giving a visual representation of their mood, and clicking the feelings gave them access to supportive emotional regulation statements Universality: the intervention messages can be linked to emphasizing common humanity Acting: users get intervention messages twice daily, and these prompt users to engage in mindfulness and self-compassion activities	
	Fonseca et al [67], 2019, Portugal	Technology: self-guided, web-based intervention (Be a Mom) Target group: at risk postpartum women and women presenting early-onset postpartum depressive symptoms Goal: to prevent persistent postpartum depression symptoms Role in intervention: stand-alone		Design: experimental (pilot RCT) Outcome measures: symptom and well-being outcomes and self-compassion		Recognition: intervention helps women to be aware of and understand their emotions and thoughts Universality: intervention offered exercises to help women accept that they are vulnerable and human like all mothers Tolerance^g^: intervention helps women to nonjudgmentally accept difficult emotions Acting: intervention helps women to use more self-compassionate ways to deal with their experiences, and addresses perinatal-specific concerns	
	Schnepper et al [68], 2020, Austria	Technology: mobile self-compassion intervention Target group: people that want to lose weight or develop healthier eating behavior Goal: to improve eating behavior, self-compassion, and stress levels Role in intervention: stand-alone		Design: experimental (RCT) Outcome measures: symptom and well-being outcomes and self-compassion		Universality: participants learned to see negative emotions as part of being human Tolerance: participants learned to be mindful and less critical about negative emotions Acting: journaling exercises explored how participants could find less critical ways to motivate themselves to improve eating behavior	
	Raymond [69], 2019, United States	Technology: SMS text messaging Target group: undergraduate psychology students Goal: to enhance psychological interventions Role in intervention: blended treatment		Design: experimental (RCT) Outcome measures: well-being outcomes		Acting: in addition to 3 daily texts with self-compassion content, participants could request additional texts to receive a recommendation to help manage strong emotions or a self-compassion quote	
	Lee et al [70], 2019, Netherlands	Technology: chatbot that was either caregiving or care-receiving (Vincent) Target group: nonclinical population Goal: to increase self-compassion Role in intervention: stand-alone		Design: experimental Outcome measures: self-compassion, opinions about the agent, inclusion of other in the self (identification with chatbot), and engagement		Universality: the care-receiving chatbot likely increased self-compassion through a substantial change in participants’ sense of common humanity	
	Köhle et al [71], 2017, Netherlands	Technology: web-based self-help intervention based on Acceptance and Commitment Therapy and self-compassion (Hold on, for each other) Target group: partners of patients with cancer Goal: to help partners to positively persevere during the difficult times they find themselves facing Role in intervention: guided		Design: qualitative Outcome measures: user experiences (appreciation of intervention and their lessons learned)		Recognition: partners learn to recognize and be aware of their own emotions Universality: partners have the option to connect with peers, eg, to share their answers on exercises and read those given by others Tolerance: partners learn how to be accepting of their difficult emotions	

^a^VR: virtual reality.

^b^Understanding the universality of suffering in the human experience.

^c^Motivation to act or acting to alleviate suffering.

^d^RCT: randomized controlled trial.

^e^Feeling empathy for the person suffering and connecting with the distress (emotional resonance).

^f^Recognizing suffering.

^g^Tolerating uncomfortable feelings aroused in response to the suffering person (eg, distress, anger, fear) so remaining open to and accepting of the person suffering.

**Table 3:** Extracted data from the included studies classified as Role C: technology supports elements of compassion between people (n=13).

Role and study			Technology (technology, target group, goal, and role in intervention)		Study (study design and outcome measures)		Compassionate elements (on the basis of the 5 elements proposed by Strauss et al [28])
	Okita [72], 2013, United States	Technology: therapeutic robot companion (Paro) Target group: pediatric patients and their parents Goal: to reduce pain and emotional anxiety in patients and their parents Role in intervention: stand-alone		Design: experimental Outcome measures: child’s pain and parent’s empathetic pain and perception of child’s pain		Recognition^a^: parents could acknowledge the patient’s pain more accurately through robot-assisted therapy Empathy^b^: robot was used as a social agent to generate perspective taking through a shared common experience and seemed to enhance parent’s ability to empathize directly with the child Acting^c^: engaging with the robot together reduced pain and emotional anxiety in the patients and reduced empathetic pain in the parents	
	Choo et al [73], 2016, United States	Technology: web-based intervention with a “booster” phone call Target group: women with coexisting intimate partner violence and substance use disorders Goal: to address violence and drug use among women patients in the emergency department Role in intervention: guided		Design: qualitative and quantitative cross-sectional Outcome measures: satisfaction, usability, and consistency with motivational interviewing		Universality^d^: technology is a link to social support, to prevent isolation Empathy: the experience of the intervention was personal and empathetic	
	Bar-Lev [74], 2008, Israel	Technology: online support group Target group: people with HIV or AIDS Goal: to provide a web-based community center, with medical information, job postings, links to community services, and a public discussion group Role in intervention: stand-alone		Design: qualitative Outcome measures: emotional dynamics in online support groups		Universality: participants in online support groups create emotionally vibrant, empathic communities by describing and sharing their experiences	
	Wijma et al [75], 2018, Netherlands	Technology: VR^e^ intervention with a VR simulation movie and e-course (Through the D’mentia Lens) Target group: informal caregivers of people with dementia Goal: to enhance understanding and empathy in caregivers of people with dementia Role in intervention: stand-alone		Design: quasi-experimental (pilot study) Outcome measures: feasibility, acceptance, caregiver’s person-centeredness, empathy, perceived pressure from care and perceived competence, and quality of relationship		Empathy: the intervention strengthened the ability of the participant to empathize with the person with dementia they take care of Tolerance^f^: and gave informal caregivers more confidence in their care task and a more positive attitude toward it by increasing their resilience	
	Han et al [76], 2011, United States	Technology: online support groups Target group: low-income women with breast cancer Goal: to help cope with illness Role in intervention: stand-alone		Design: qualitative Outcome measures: empathy and emotional support expression and reception and breast cancer–related concerns		Universality: online support groups provide a community to connect with people going through similar experiences Empathy: online support groups provide patients with a space to share illness experiences, feelings, and concerns, and these self-disclosing activities stimulate empathic responses from others Acting: participants can read and write empathic messages to respond compassionately to each other’s distress, and this seems to reduce breast cancer concerns	
	Högberg et al [77], 2018, Sweden	Technology: web-based communication service Target group: patients with hematological diseases Goal: to offer patients the possibility to request support from a nurse Role in intervention: telecommunication		Design: qualitative Outcome measures: nurse’s abilities of compassion, competence, and upholding trust		Recognition: patients can share personal everyday experiences and their worries, and the nurse can explicitly express recognition Empathy: the nurse can express caring and interest to respond compassionately and reflect the tone of the message Tolerance: the nurse can respond in a supportive way by expressing acceptance and validation Acting: patients can explicitly request for direct actions, which are achieved because of the communication rather than via the communication per se	
	Steinwachs et al [78], 2011, United States	Technology: interactive web-based intervention (YourSchizophreniaCare) Target group: patients with schizophrenia Goal: to help patients to discuss their mental health treatment with their therapist Role in intervention: stand-alone		Design: experimental Outcome measures: client’s and clinician’s respective contributions to dialogue and clinician’s empathy		Recognition: patients answer questions about their current status and treatment, eg, how often their medication makes them restless Acting: on the basis of their answers, they can get recommendations to discuss a topic with the therapist, and feedback on how to do it effectively	
	Kysely et al [79], 2020, Australia	Technology: videoconferencing Target group: couples Goal: to deliver psychotherapy, specifically relationship interventions Role in intervention: telecommunication		Design: qualitative Outcome measures: expectations and experiences with videoconferencing, ie, empathy		Empathy: ambiguous; the distance between therapist and client in videoconferencing can stimulate the client to open up more because of feeling safe and empowered, but it can also be experienced as impersonal	
	Blair Irvine et al [80], 2012, United States	Technology: web-based training program, multimedia (Caring Skills: working with Mental Illness) Target group: licensed care staff working in long-term care facilities Goal: to increase empathy and decrease stigmatization toward residents with mental illness Role in intervention: telecommunication		Design: experimental (RCT^g^) Outcome measures: care staff member’s self-efficacy, attitudes toward people with mental illnesses, behavior intentions, empathy, acceptance, and usability		Empathy: intervention stimulates engaging the resident in conversation to attempt to understand their perspective, listening to them and acknowledging their emotion Tolerance: intervention conveys a person-centered care philosophy, where resident’s potentially problematic behavior is seen as an expression of an unmet need, rather than just behavior to be managed, and promotes self-care for the caregiver after upsetting interactions with residents Acting: training includes behavioral skills to work with mental illness behavior, such as acknowledging the resident’s emotions and finding a suitable redirection	
	van Rijn et al [81], 2017, United Kingdom	Technology: VR-on a laptop, not “immersive” (ProReal) Target group: prisoners in a therapeutic community prison Goal: to improve mental health outcomes and mental well-being, as an addition to the existing therapeutic intervention Role in intervention: blended treatment		Design: qualitative and quasi-experimental Outcome measures: symptom and well-being outcomes, engagement, and quality of relationships between participants and with counselor		Recognition: the program enables clients to make their thoughts, feelings, and experiences visible in a web-based representation with avatars, supporting their self-expression and understanding Empathy: the program helps to reflect on situations and experiences and to see them from different perspectives that clients could empathize with	
	Fordham and Ball [82], 2019, United States	Technology: digital game (Hellblade: Senua’s Sacrifice) Target group: general public Goal: to create embodied experiences of mental health and promote empathic understanding (in this case focused on representations of psychosis) Role in intervention: stand-alone		Design: qualitative Outcome measures: the design of embodied experiences of mental illness		Empathy: the game allows the player to experience symptoms similar to a psychosis (eg, auditory and visual hallucinations)	
	Tippin and Maranzan [83], 2019, Canada	Technology: web-based antistigma video intervention (Photovoice) Target group: general public Goal: to reduce mental illness stigma Role in intervention: stand-alone		Design: experimental (RCT) Outcome measures: stigma toward mental illness and empathic concern		Empathy: intervention conveys lived experiences with mental illness and evokes empathic concern in watcher Tolerance: intervention led to decreased anger and fear toward people with a mental illness	
	Milbury et al [84], 2020, United States	Technology: web-based meditation intervention for couples via FaceTime (led by counselor) Target group: patients with primary and metastatic brain tumors and their partners Goal: to target symptom and well-being outcomes Role in intervention: telecommunication		Design: experimental (pilot RCT) Outcome measures: symptoms and well-being outcomes, compassion in couples’ relationship, and feasibility		Recognition: couples were introduced to meditation techniques by guiding their awareness to their current experience Empathy: couples participated in meditations that focused their attention on their interconnectedness and their feelings of compassion for the self and the partner Tolerance: participants were asked to share experiences with each other in a state of nonjudgmental and accepting awareness	

^a^Recognizing suffering.

^b^Feeling empathy for the person suffering and connecting with the distress (emotional resonance).

^c^Motivation to act or acting to alleviate suffering.

^d^Understanding the universality of suffering in the human experience.

^e^VR: virtual reality.

^f^Tolerating uncomfortable feelings aroused in response to the suffering person (eg, distress, anger, fear) so remaining open to and accepting of the person suffering.

^g^RCT: randomized controlled trial.

### Technologies’ Types, Goals, Target Groups, and Roles in the Intervention

To answer the first research question, we will describe the different types, goals, target groups, and roles in technological interventions for each compassionate role. Table 4 shows a numeric summary of the results, including the frequencies and references of the variables that had a limited number of clear subtypes (technology types, roles in intervention, and compassionate elements). In Role A, we found 8 technologies showing elements of compassion for a person. An example is a chatbot that replies empathically to the person’s input and motivates them to complete certain activities, such as modules based on CBT [52]. For this role, the types of technology were mainly chatbots or conversational agents [52,53,57,58] and social robots [54,56,59]. The most identified goal was for technology to express empathic support and to foster the therapeutic alliance between the person and technology [52,53,55,58]. The target groups included different groups of people, such as people with dementia [59], schizophrenia [58], and ongoing or cyclic pain [52]. Some target groups were more general, such as “people who have difficulty living with pets” [56], or “people filling in the Beck’s Depression Inventory” [55]. For Role A, we found almost exclusively stand-alone interventions [52-58], meaning that they were used by the target group without any guidance from a coach or therapist. One exception was a social robot used in blended treatment [59], where the robot functioned as an assistant to the therapist in psychomotor therapy.

**Table 4:** Numerical summary of the extracted variables that had a limited number of clear subtypes, including their frequencies and references, per compassionate role.

Compassionate role, variable, and subtypes				Frequency, n (%)		References
**Role A (n=8)**						
	**Technologies**					
		Chatbot or conversational agent	4 (50)		[52,53,57,58]	
		Social robot	3 (38)		[54,56,59]	
		Web-based agent	1 (12)		[55]	
	**Roles in interventions**					
		Stand-alone	7 (88)		[52-58]	
		Blended treatment	1 (12)		[59]	
	**Compassionate elements**					
		Recognition	4 (50)		[52,54,57,59]	
		Universality	N/A^a^		N/A	
		Empathy	8 (100)		[52-59]	
		Tolerance	N/A		N/A	
		Acting	5 (63)		[52,54,57-59]	
**Role B (n=12)**						
	**Technologies**					
		Virtual reality	4 (33)		[60,61,63,64]	
		Mobile phone app or SMS text messaging	4 (33)		[65,66,68,69]	
		Web-based intervention	3 (25)		[62,67,71]	
		Chatbot	1 (8)		[70]	
	**Roles in interventions**					
		Stand-alone	10 (83)		[60-64,66-68,70]	
		Guided	1 (8)		[71]	
		Blended treatment	1 (8)		[69]	
	**Compassionate elements**					
		Recognition	4 (33)		[65-67,71]	
		Universality	7 (58)		[60,65-68,70,71]	
		Empathy	2 (17)		[61,62]	
		Tolerance	3 (25)		[67,68,71]	
		Acting	9 (75)		[60,61,63-69]	
**Role C (n=13)**						
	**Technologies**					
		Web-based intervention	5 (38)		[73,77,78,80,83]	
		Online support group	2 (15)		[74,76]	
		Videoconferencing	2 (15)		[79,84]	
		Virtual reality	2 (15)		[75,81]	
		Social robot	1 (8)		[72]	
		Digital game	1 (8)		[82]	
	**Roles in interventions**					
		Stand-alone	7 (54)		[72,74-76,78,82,83]	
		Telecommunication	4 (31)		[77,79,80,84]	
		Guided	1 (8)		[73]	
		Blended treatment	1 (8)		[81]	
	**Compassionate elements**					
		Recognition	5 (38)		[72,77,78,81,84]	
		Universality	3 (23)		[73,74,76]	
		Empathy	11 (85)		[72,73,75-77,79-84]	
		Tolerance	5 (38)		[75,77,80,83,84]	
		Acting	5 (38)		[76-78,80]	

^a^N/A: not applicable.

In Role B, we found 12 technologies that were used to enhance the elements of self-compassion in the person using them. An example is a VR intervention set in space to support the development of self-compassion [60]. The most frequently used technology types are VR [60,61,63,64], mobile phone apps, and SMS text messaging [65,66,68,69]. The goals mentioned most often for technologies with this role were to enhance self-compassion [60,62-64,66,68,70] and to decrease psychological symptoms [61,62,67,68]. Target groups were sometimes specific, for example, people with paranoia symptoms [60,61], and sometimes broader, for example, adolescents [65,66]. For Role B, most technologies served as a stand-alone intervention [60-68,70]. We found 1 study in which the intervention was guided in the form of written feedback from a personal web-based counselor [71]. Finally, in 1 study, the technology was part of a blended treatment [69]. Here, SMS text messaging was used to enhance a physical psychological intervention on self-compassion [69].

In Role C, we found 13 technologies that were used to facilitate elements of compassion between people, for instance, a therapeutic robot that acts as a social agent between pediatric patients and their parents in robot-assisted therapy [72]. The types of technologies were mainly web-based interventions [73,74,76-78], online support groups [74,76], videoconferencing [79,84], and VR [75,81]. Various goals were found, these included decreasing psychological symptoms [72,73,81,84] and enhancing empathy [75,80,82]. Target groups were often people with physical illnesses [72,74,76,77,84] but also included informal caregivers [75,84] and licensed care staff [80]. For Role C, again, most technologies formed a stand-alone intervention [72,74-76,78,82,83]. We also found several technologies that were used for telecommunication [77,79,80,84]. We found 1 study where the technological intervention was guided (albeit minimally), pertaining to the use of a “booster phone call” to review the process and challenges [73]. Finally, we also found 1 study in which technology was part of blended treatment: a VR intervention that was used in physical sessions with a counselor [81].

### Study Designs and Outcome Measures

A range of different study designs and outcome measures was found in the included studies. Regarding outcome measures, none of the studies evaluated whether the technology as such was (perceived to be) compassionate. For the 8 articles categorized as Role A (technology showing compassion), we found the following study designs: experimental [52,57,59], quasi-experimental [55,58], qualitative [54], a combination of qualitative and experimental [53], and a combination of quantitative cross-sectional and qualitative [56]. Outcome measures included the acceptability of the intervention [52,56,57], evaluations of the technology [53,55,59], and symptoms and well-being [52,57,59]. One study investigated empathy in human-robot interaction by observing people with dementia interacting with a therapy robot during psychomotor therapy [59].

For the 12 articles categorized as Role B (enhancing self-compassion), the study designs were almost exclusively experimental [60-62,64,66-70], followed by qualitative [71] and quasi-experimental [63]. All but 1 study used specific outcome measures, most often symptoms and well-being [60-64,66-69] and self-compassion [60-63,66-68,70].

Finally, for the 13 articles categorized as Role C (facilitating compassion), the study designs were mostly qualitative [74,76,77,79,82] or experimental [72,78,80,83,84], followed by quasi-experimental [75], a combination of qualitative and quantitative cross-sectional [73], and a combination of qualitative and quasi-experimental [81]. Found outcome measures were mostly empathy between humans [72,75,76,78-80,83] and symptoms and well-being [72,76,81,84]. One study had compassion as an outcome measure, referring to nurses’ abilities to be compassionate through web-based communication [77].

### Elements of Compassion

#### Overview

In general, empathy was used slightly more often as the main term to describe certain elements of technologies in the included studies (18/33, 55%) than compassion (15/33, 45%). Compassion was most often found in the form of increasing self-compassion (Role B). We did not find any technologies that embedded all the 5 elements of compassion. In the following sections, we will further specify how the 5 elements were embedded in the included DMHIs.

#### Recognition

The first element of compassion proposed by Strauss et al [28] is the recognition of suffering. This was observed in 13 of the included studies. In Role A (technology showing compassion), recognition referred to users being able to indicate their thoughts and feelings toward the technology. For example, people could indicate their mood to a fully automated conversational agent and receive a weekly mood description [57]. Similarly, in Role B (enhancing self-compassion), technology could allow users to track their feelings daily in a mood cloud, prompting personalized feedback or supportive emotional regulation statements to enhance self-compassion [66]. In Role C (facilitating compassion), technology could be used to help caregivers recognize suffering. An example is a robot seal that helped parents to acknowledge their children’s pain more accurately in robot-assisted therapy [72]. In another study, people with schizophrenia could use a web-based application to answer questions about their status and treatment, so the app could then act by giving recommendations on what they could discuss with their therapist and how [78]. Thus, unsuitable medications or other issues that could arise could be recognized faster.

#### Universality

This element refers to the understanding of the universality of suffering in human experiences [28]. It was only found in 10 of the included studies, of which none belonged to Role A, 7 studies were categorized as Role B, and 3 as Role C. In Role B, the technology could give shape to universality by giving user messages or exercises that emphasize common humanity, and that negative emotions are part of being human. For instance, in 1 study, women at risk for postpartum depressive symptoms received exercises in a web application to help them accept that they are susceptible and human, similar to all mothers [67]. Another study involved the chatbot Vincent, who “talked” about his mistakes, supporting common humanity [70]. In Role C, technology supported universality by providing a link to social support [73] so that people could exchange experiences and prevent isolation.

#### Empathy

This element refers to feeling empathy for those who are suffering and connecting with distress (emotional resonance [28]). Overall, the element “empathy” was the most frequently found (n=29). For Role A, empathy was found in all the studies (n=8). Most studies simply mentioned that the technology used an empathic tone in the messages it sent [52]; in some cases, it was tailored to the user’s input (current mood or text). In Role B, the element of empathy was found only twice. It refers to technology fostering the user’s empathy with the goal of targeting symptoms and well-being outcomes by letting them imagine an unpleasant experience they had from the perspective of an observer [62]. The other time it was found in a study where participants created a personal compassionate coach in VR that provided kindness and warmth, to help them be more self-compassionate [61]. In Role C, technology supports empathy among people in diverse ways and was found 11 times. Usually, technology facilitates empathy between a client and an informal or formal caregiver, for example, through a VR intervention that allows informal caregivers to experience dementia [75]. Technology can also facilitate empathy between a client and other people with similar experiences, for example, through an online support group [76]. Finally, technology could foster empathy in the general public for those with mental illnesses [83].

#### Tolerance

The element that was found least in the included studies was “tolerance” (n=8), referring to tolerating uncomfortable feelings aroused in response to the suffering person (such as distress, anger, or fear) and remaining open to and accepting of the person suffering [28]. Tolerance was not found in Role A. For Role B, tolerance was found 3 times. In all cases, it referred to supporting tolerance in humans and was described as helping people to be nonjudgmental and accepting toward themselves [68]. Finally, for Role C, tolerance was found the most frequently (n=5). In one instance, it was expressed in a VR intervention, giving informal caregivers of people with dementia more confidence in their care tasks and a more positive attitude toward them by increasing their resilience and proactive competence [75]. Similarly, a web-based training program for licensed care staff conveyed the philosophy that a resident’s potentially problematic behavior is an expression of an unmet need, instead of just behavior that needs to be managed [80]. In another study, tolerance was mentioned more indirectly, stating that a web-based communication service allowed a nurse to respond by expressing acceptance and validation [77].

#### Acting

Finally, the element “acting” refers to the motivation to act or actually acting to alleviate suffering [28]. This element was identified in 20 of the included studies. In Role A, acting was found 6 times. It was often found in very practical ways, stimulating the user to complete activities [52]. In some cases, the prompted content or activities were offered by the technology based on the user’s input, such as their mood state [57] or medication-taking behavior [58]. In one case, a social robot was used to provide positive feedback and motivate a person with dementia to engage in psychomotor therapy with their therapist [59]. For Role B, acting was found 9 times, where the technology acted to enhance the user’s self-compassion. For instance, VR was used to allow people to interact with a space nebula representing compassion, guiding them to open up compassionate feelings to increase self-compassion and decrease paranoia [60]. In another case with VR, people delivered compassionate sentences to a virtual child and then experienced these again from the perspective of the child [64]. To facilitate compassion in Role C, acting was found 5 times and could take different shapes. Technology could act as a social agent to stimulate empathic social interactions with other people [72]. Other times, the element of acting referred to human action facilitated by technology, such as the possibility of requesting support from a nurse [77].

## Discussion

### Principal Findings

This systematic scoping review investigated how and to what extent technology for mental health care has been connected to compassion in previous research. We could identify 3 roles that technology can play to support compassion in mental health care: showing compassion *to* the client, enhancing self-compassion *in* people, and facilitating compassion *between* people, such as between a client and a caretaker or therapist. The main types of technologies and their goals, as well as the main study designs and outcome measures, differed by role, whereas the main target groups varied widely across all roles. We found a large majority of technologies that were described as stand-alone interventions, to be used by the target group without guidance from a coach or therapist. Only a few included studies described technologies as part of blended treatment: integrated in face-to-face sessions with a coach or therapist. This is in line with earlier research showing a lack of clarity on how to embed technologies in blended treatment [19]. Furthermore, none of the technologies included all elements of a proposed definition of compassion [28], nor was any technology evaluated on whether it was experienced as compassionate. However, we recognized certain elements of compassion in the technologies, showing that all elements could potentially be embedded in technology.

Of the 5 proposed elements of compassion, we found descriptions matching the elements “empathy” and “acting” most often in the included studies, followed by “recognition.” In the scoping review care by Kemp et al [37], DMHIs were examined using a model of digital intersections with compassionate care. The authors found DMHIs that could support 4 of the 6 categories in this model. However, for 2 categories, no DMHIs were found in their review. These were “awareness of suffering” (the use of a DMHI to become more aware of one’s suffering) and “mediated response” (the use of a DMHI to mediate the response to suffering) [37]. “Awareness of suffering” seems to be related to “recognition of suffering” in our current review, whereas “mediated response” could have overlap with “acting to alleviate suffering.” We found several examples of how DMHIs could support both compassion components.

The elements of “universality” and “tolerance” were rarely found “Universality” was mostly found in the studies in Role B, describing DMHIs with the aim to enhance self-compassion. These studies often followed the definition of self-compassion by Neff [29], which overlaps with the definition of compassion proposed by Strauss et al [28]. Furthermore, as mentioned before, none of the included studies described technologies that fostered all elements of compassion. As Sinclair et al [38] discussed in their review on compassion in health care, the separate elements of compassion are not inherently compassionate; rather, their combination constitutes compassion. Following this logic, combined with the lack of evaluation of technology on (perceived) compassion, we cannot be sure if truly compassionate technology exists within our scope.

### The Added Value of Compassion

Empathy was the element of compassion that we identified most frequently and that was used more often as a main term to describe technologies than compassion itself. As discussed in the Introduction section, there are some important differences between the constructs [28,33]. In short, compassion is specifically a response to suffering, whereas empathy can be felt for any emotion. Further, compassion includes the motivation to act to alleviate suffering, whereas empathy can also be followed by behavior in general but does not necessarily include this. These differences, as well as the additional elements of understanding the universality of human suffering and distress tolerance, make compassion especially valuable in the field of mental health or mental health care [37]. Therefore, we believe that explicitly embedding elements of compassion in the design and use of technology for mental health care, not just empathy, is a promising approach that is currently lacking.

In the current review, the lesser-known elements of compassion that are just as important in mental health care, such as understanding the universality of suffering in human experience and tolerating uncomfortable feelings, were rarely found. This indicates that a too narrow (or no) definition of compassion is used, at least when referring to features of technology. The overarching construct of compassion in “traditional” mental health care is a fundamental value and has multiple beneficial effects [28,37]. Moreover, considering the intentions, motivations, and values of stakeholders in mental health care or health care is argued to be essential in successfully blending technology into this field [12,37,85]. Thus, considering the elements of compassion in the design and use of future digital interventions could be a promising approach to improving the acceptance of DMHIs in the mental health context.

### The Potential of Compassionate Technology

Our findings show that compassion offers a versatile and potentially transdiagnostic lens with which to examine technology for mental health care. First, as discussed in the Introduction, mental health professionals expressed the need for a clear conceptual basis for embedding technologies into mental health care [19]. Compassion could be a suitable basis and guiding force for the integration of technologies in mental health care: blending in technology in such a way that the therapeutic process as a whole is optimally in line with all elements of compassion. Thus, recognizing and alleviating suffering (ie, compassion) would be the central goal of both the design and evaluation of DMHIs as well as protocols for working with technology. Conventional values such as efficiency or effectiveness would be considered as means to achieve this goal. The design methodology of “Values that Matter” would lend itself very well to the design of DMHIs around compassion, as it aims to embed ethical values in technology [86].

**Figure 3:**
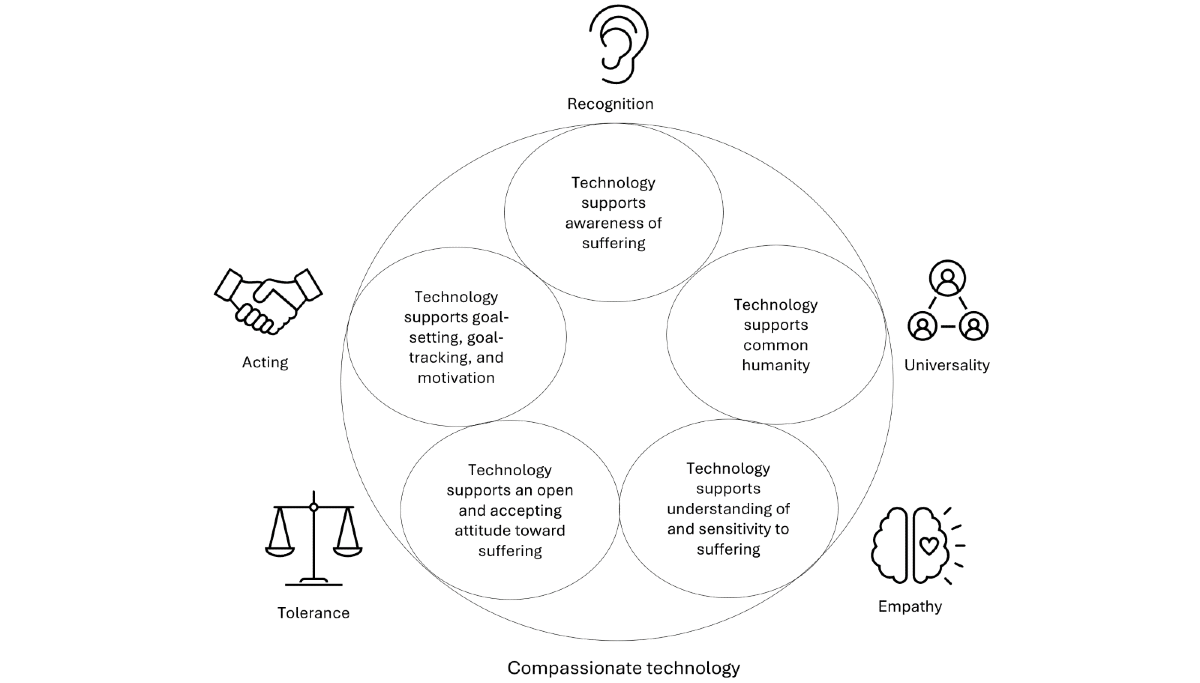
Conceptual overview of compassionate technology, which supports all elements of compassion.

Second, not only can compassion offer the basis for the design of DMHIs and protocols for the integration of compassionate technology in treatment, but the other way around technology can also bring new and additional ways to foster compassion that are not possible in traditional treatment (see Figure 3 for a conceptual overview). Examples include being present anywhere and anytime without getting tired or frustrated or being easier and more accessible to share suffering. On a microlevel, every interaction with a DMHI could convey all elements of compassion, and the elements of compassion could also be conveyed in the treatment as a whole by the therapist and technology together or at different times during the therapeutic process. Here, we illustrate what the latter could look like with an extended treatment scenario based on the 5 proposed elements of compassion [28] and the elements of compassion found in the different technologies included in our review. We also referred to relevant related initiatives that did not fully meet our inclusion criteria and were therefore excluded but still provided interesting additional possibilities.

First, the DMHI could help the client and therapist to (1) *recognize* moments of suffering anytime and anywhere. This could be based on input from the client (eg, indicating their mood and feelings [57]) but could also be combined with physiological measurements from a wearable (an example can be found in Fletcher et al [87]). The DMHI could convey the (2) *universality* of suffering in the human experience, for example, by connecting the client to similar experiences of others [73]. Furthermore, the DMHI could support the presence of (3) *empathy* for clients’ suffering by sending empathic messages based on the user’s input [52] or by helping the therapist to offer emotional support specifically during moments of potential distress [77].

Furthermore, the tone in communication with the DMHI and the therapist would remain open, accepting, and (4) *tolerant* of distress by acknowledging and validating difficult feelings (eg, [67,88]), where the DMHI has the advantage that it is not susceptible to empathic distress and can always be there for the client when it is needed. DMHIs could also be used to help increase the resilience and distress tolerance of mental health professionals [80]. Finally, together with the therapist, the client could look back on the recorded and experienced difficult moments and successes and set therapeutic goals. The DMHI could help to keep track of and work on these goals [57], until the next appointment with the therapist, for instance, by activating tailored exercises at the right moment and giving reminders or rewards to support (5) *acting* to alleviate suffering. Of course, this example raises new questions that need to be explored, such as how the tasks of the therapist and technology can be optimally blended to offer all the elements of recognizing and relieving suffering in an iterative manner.

### Critical Notes on Digital Environments in Relation to Compassion and Empathy

This review has shown the possibilities for DMHIs to contribute to compassion in the field of mental health in several ways. However, it is important to acknowledge that concerns also exist regarding the use of technology in (mental) health care. First, there is concern about technology detracting from empathy and compassion. This could happen because of a lack of emotional signals and cues, and the possibility of hiding behind anonymity, and easily escaping the reactions of others [37,89]. In our review, we saw this in an article describing ambiguous experiences with videoconferencing, which could be considered impersonal [79]. These concerns point to the importance of placing more emphasis on empathy and compassion if DMHIs are used, such as by preparing (mental) health professionals to use DMHIs in a compassionate way for both themselves and their clients [89]. One possible way to enhance empathy among health professionals could be VR interventions [90,91]. In addition, there is debate about whether DMHIs actually contribute to equal access to care or whether they enlarge the existing inequities in society. For example, groups that have limited access to digital health care and limited digital literacy skills could be left behind [92]. These concerns should be considered in the development, evaluation, and use of DMHIs in mental health care. For instance, future evaluation of DMHIs should critically assess for the presence and quality of different compassion elements.

### Limitations

This review is a systematic scoping review into an unexplored and diverse field. As we searched for mentions of compassion or empathy to delimit our research area, there could be technologies for mental health care that we did not include here but do show elements of compassion. There are several related initiatives in technology development, such as calm technology and affective computing, which focus on unobtrusive and emotion-sensitive technologies, respectively [93,94]. However, because this scoping review aims to provide an overview of technology explicitly related to compassion or empathy, these related initiatives did not fall within our scope.

Furthermore, it is important to keep in mind that our conceptualization of compassion, and especially the acceptability and value of suffering within Western psychological theories and therapies, may have cross-cultural limitations [95,96]. As the majority of studies included in the current review were conducted in Western countries, our findings may represent a mostly Western view of compassion and suffering. For instance, non-Western cultures generally do not see suffering as a purely negative life experience but instead believe that by appreciating it for what it offers, it can actually contribute to living a good life [95].

In addition, because the term “compassionate technology” was not found in previous studies, we used the definition of compassion proposed by Strauss et al [28] to assess the extent to which the technology in the included studies matched elements of compassion. In doing so, we closely followed the phrasing of the study authors. However, authors often did not explain (in detail) what they understood as “empathic” or “compassionate.” Therefore, we may have misinterpreted the meaning of the authors. To make future studies on compassionate technology more transparent and comparable, we recommend that the authors include their definitions of compassion or its elements.

Because the present review was a scoping review, we did not assess the methodological quality of the included articles but instead focused on creating an overview of the scope of this field. Moreover, although some studies have measured compassion or self-compassion as an outcome, no studies have measured how compassionate the technology was, for example, as experienced by its users. We do not know whether the compassionate elements actually contributed to the presence of compassion, and if so, how and which elements did. Thus, we cannot be sure that the technologies we included measurably showed, enhanced, or facilitated compassion in the mental health care process.

### Future Research

On the basis of the increasing frequency we saw over the publication years of the included articles linking compassion and empathy to DMHIs, the attention for such values in this field seems to be growing. This makes it a promising area for further research, but also one where much remains to be discovered. Most importantly, compassion is not yet seen as a foundation and goal for embedding technology into mental health care, and research is needed to learn more about how to design technologies and blended ways of working around compassion, focusing on the optimal recognition and alleviation of suffering. Furthermore, no scale exists to evaluate DMHIs on compassion. To advance the field of compassionate technology, it is essential to be able to determine which types of technologies with which design features actually support the presence of compassion across the 3 roles and for whom. Thus, a scale needs to be developed to measure compassion as shown, enhanced or facilitated by technology for both clients and professionals in mental health care.

Finally, as a scoping review, this review focused on the scope and degree to which we could find compassion linked to DMHIs but did not consider the effects of technology with compassionate elements on, for example, adherence [97], engagement [98], or effectiveness. Future research is needed to study which validated measures have been used in this field, so that the effects on the aforementioned constructs could be researched. Although the types of research and outcome measures we found varied widely, research on technology enhancing self-compassion *in* people consisted mainly of experimental studies measuring participants’ psychological symptoms or self-compassion or both. Therefore, research in this role would lend itself well to a meta-analysis of the effectiveness of these technologies. Moreover, we used search terms related to mental health but found many studies related to mental well-being, with some components of mental health. Thus, it could be interesting for future research to expand the scope to explicitly include DMHIs around mental well-being or lifestyle, because there might be additional relevant work in these fields as well.

### Conclusions

Compassion is an essential value in mental health care, pertaining to recognizing suffering, being moved by it, and acting to alleviate it. Given the importance and benefits of compassion in mental health care, shifting the focus of the design, evaluation, and use of DMHIs to center on compassion seems to be a new, fascinating, and perhaps even necessary direction in research and clinical practice. This scoping review explored how and to what degree elements of compassion have been linked to technologies for mental health care in previous studies. Our review shows that compassion is a widely applicable construct across different technologies, target groups, and for different aims in mental health care and is potentially a guiding force in embedding technology in mental health care. Moreover, it provides new input for the design and development of technology around compassion and demonstrates the necessity of evaluating technology on this foundational value in mental health care. Overall, this review serves as a first step toward “compassionate technology” as a guiding principle in the use and design of technology for mental health care. This principle refers to technology that contributes to the recognition and alleviation of suffering and is appropriately suited to the mental health care context for both clients and professionals.

## Appendix

Multimedia Appendix 1 Search strategies for each of the included databases.

## Abbreviations

CBT: cognitive behavioral therapy
DMHI: digital mental health intervention
VR: virtual reality
