# Supplementary material for: The Role of Compassionate Technology in Blended and Digital Mental Health Interventions: Systematic Scoping Review
Source: JMIR Ment Health. 2023 Apr 7;10:e42403. doi: 10.2196/42403 (PMC10131870; doi:10.2196/42403)
Supplement: Multimedia Appendix 1 [file mental_v10i1e42403_app1.docx]

## Appendix

Table 2. Search strategies for each of the included databases.

| **Pubmed**  191 results | ("compass*"[Title/Abstract] OR "empath*"[Title/Abstract] OR "empathy"[MeSH Terms]) AND ("mental"[Title/Abstract] OR "emental"[Title/Abstract] OR "e-mental"[Title/Abstract] OR "telementalhealth"[Title/Abstract] OR "psycholog*"[Title/Abstract]) AND ("ehealth"[Title/Abstract] OR "e-health"[Title/Abstract] OR "remote"[Title/Abstract] OR "telemedicine"[MeSH Terms] OR telemedicine[Text Word] OR "telehealth"[Title/Abstract] OR "mhealth"[Title/Abstract] OR "serious games"[Title/Abstract] OR "affective computing"[Title/Abstract] OR "positive computing"[Title/Abstract] OR "biosensing"[Title/Abstract] OR "biofeedback"[Title/Abstract] OR "blended"[Title/Abstract] OR "technology-mediated"[Title/Abstract] OR "technology-mediated"[Title/Abstract] OR "digital"[Title/Abstract] OR "internet"[Title/Abstract] OR "online"[Title/Abstract] OR "web-based"[Title/Abstract] OR "mobile"[Title/Abstract] OR "vr"[Title/Abstract] OR "virtual reality"[Title/Abstract] OR "ai"[Title/Abstract] OR "artificial intelligence"[Title/Abstract] OR "robot*"[Title/Abstract]) AND ("intervention"[Title/Abstract] OR "therap*"[Title/Abstract] OR "Psychotherapy"[Mesh] OR "treatm*"[Title/Abstract]) |
| --- | --- |
| **Scopus**  310 results | TITLE-ABS-KEY ( ( compass*  OR  empath* )  AND  ( "emental health"  OR  "e-mental health"  OR  telementalhealth  OR  ( ( mental  W/5  ( ehealth  OR  e-health OR  telemedicine  OR  telehealth  OR  mhealth  OR  "serious games"  OR  "affective computing"  OR  "positive computing"  OR  biosensing  OR  biofeedback ) ) )  OR  ( ( blended  OR  technology-mediated  OR  "technology mediated"  OR  digital  OR  internet  OR  online  OR  web-based  OR  mobile  OR  vr  OR  "virtual reality"  OR  ai  OR  "artificial intelligence"  OR  robot OR remote)  W/5  ( intervention  OR  therap*  OR  treatm* ) ) ) ) |
| **PsycInfo**  152 results | TI ( (( compass* OR empath* ) AND ("emental health" OR "e-mental health" OR telementalhealth OR ( ( mental N5 ( ehealth OR e-health OR telemedicine OR telehealth OR mhealth OR "serious games" OR "affective computing" OR "positive computing" OR biosensing OR biofeedback ) ) ) OR ( ( blended OR technology-mediated OR "technology mediated" OR digital OR internet OR online OR web-based OR mobile OR vr OR "virtual reality" OR ai OR "artificial intelligence" OR robot OR remote ) N5 ( intervention OR therap* OR treatm* ) ) ) ) )  OR AB ( (( compass* OR empath* ) AND ("emental health" OR "e-mental health" OR telementalhealth OR ( ( mental N5 ( ehealth OR e-health OR telemedicine OR telehealth OR mhealth OR "serious games" OR "affective computing" OR "positive computing" OR biosensing OR biofeedback ) ) ) OR ( ( blended OR technology-mediated OR "technology mediated" OR digital OR internet OR online OR web-based OR mobile OR vr OR "virtual reality" OR ai OR "artificial intelligence" OR robot OR remote ) N5 ( intervention OR therap* OR treatm* ) ) ) ) )  OR KW ( (( compass* OR empath* ) AND ("emental health" OR "e-mental health" OR telementalhealth OR ( ( mental N5 ( ehealth OR e-health OR telemedicine OR telehealth OR mhealth OR "serious games" OR "affective computing" OR "positive computing" OR biosensing OR biofeedback ) ) ) OR ( ( blended OR technology-mediated OR "technology mediated" OR digital OR internet OR online OR web-based OR mobile OR vr OR "virtual reality" OR ai OR "artificial intelligence" OR robot OR remote ) N5 ( intervention OR therap* OR treatm* ) ) ) ) ) |
| **Web of science**  247 results | TS= (( compass* OR empath* ) AND ("emental health" OR "e-mental health" OR telementalhealth OR ( ( mental NEAR/5 ( ehealth OR e-health OR telemedicine OR telehealth OR mhealth OR "serious games" OR "affective computing" OR "positive computing" OR biosensing OR biofeedback ) ) ) OR ( ( blended OR technology-mediated OR "technology mediated" OR digital OR internet OR online OR web-based OR mobile OR vr OR "virtual reality" OR ai OR "artificial intelligence" OR robot OR remote) NEAR/5 ( intervention OR therap* OR treatm* ) ) ) ) |
